# The global burden of trichiasis in 2016

**DOI:** 10.1371/journal.pntd.0007835

**Published:** 2019-11-25

**Authors:** Rebecca M. Flueckiger, Paul Courtright, Mariamo Abdala, Amza Abdou, Zaid Abdulnafea, Tawfik K. Al-Khatib, Khaled Amer, Olga Nelson Amiel, Sossinou Awoussi, Ana Bakhtiari, Wilfried Batcho, Assumpta Lucienne Bella, Kamal Hashim Bennawi, Simon J. Brooker, Brian K. Chu, Michael Dejene, Djore Dezoumbe, Balgesa Elkheir Elshafie, Aba Ange Elvis, Djouma Nembot Fabrice, Fatma Juma Omar, Missamou François, Drabo François, Jambi Garap, Michael Gichangi, André Goepogui, Jaouad Hammou, Boubacar Kadri, George Kabona, Martin Kabore, Khumbo Kalua, Mathias Kamugisha, Biruck Kebede, Kaba Keita, Asad Aslam Khan, Genet Kiflu, Makoy Yibi, Garae Mackline, Colin Macleod, Portia Manangazira, Michael P. Masika, Marilia Massangaie, Takafira Mduluza, Nabicassa Meno, Nicholas Midzi, Abdallahi Ould Minnih, Sailesh Mishra, Caleb Mpyet, Nicholas Muraguri, Upendo Mwingira, Beido Nassirou, Jean Ndjemba, Cece Nieba, Jeremiah Ngondi, Nicholas Olobio, Alex Pavluck, Isaac Phiri, Rachel Pullan, Babar Qureshi, Boubacar Sarr, Do Seiha, Gloria Marina Serrano Chávez, Shekhar Sharma, Siphetthavong Sisaleumsak, Khamphoua Southisombath, Gretchen Stevens, Andeberhan Tesfazion Woldendrias, Lamine Traoré, Patrick Turyaguma, Rebecca Willis, Georges Yaya, Souleymane Yeo, Francisco Zambroni, Jialiang Zhao, Anthony W. Solomon

**Affiliations:** 1 Faculty of Infectious and Tropical Diseases, London School of Hygiene & Tropical Medicine, London, United Kingdom; 2 Kilimanjaro Centre for Community Ophthalmology, Division of Ophthalmology, University of Cape Town, Cape Town, South Africa; 3 Ministerio da Saude, National Ophthalmology Program, Maputo, Mozambique; 4 Ministère de la Santé et de la population, Niamey, Niger; 5 Ministry of Health, Baghdad, Iraq; 6 National Eye Health Programme, Ministry of Public Health and Population, Sana’a, Yemen; 7 Ministry of Health, Cairo, Egypt; 8 Ministerio da Saude, Maputo, Mozambique; 9 Ministère de la Santé, Lomé, Togo; 10 The Task Force for Global Health, Atlanta, GA, USA; 11 Ministère de la Santé, Cotonou, Benin; 12 Ministère de la Santé, Yaoundé, Cameroun; 13 Prevention of Blindness Program, Khartoum, Sudan; 14 Global Health, Neglected Tropical Diseases, Bill & Melinda Gates Foundation, Seattle, USA; 15 Michael Dejene Public Health Consultancy Services, Addis Ababa, Ethiopia; 16 Ministère de la Santé, N'Djamena, Chad; 17 Trachoma Control Program, National Ministry of Health, Khartoum, Sudan; 18 Programme National de la Santé Oculaire et de la lutte contre l'Onchocercose, Abidjan, Côte d'Ivoire; 19 Ministry of Health, Zanzibar, United Republic of Tanzania; 20 Bureau des Maladies Oculaires, Ministère de la Santé, Kinshasa, Democratic Republic of the Congo; 21 Programme national de lutte contre les maladies tropicales négligées (PNMTN), Ouagadougou, Burkina Faso; 22 National Department of Health, Port Moresby, Papua New Guinea; 23 Ministry of Health, Nairobi, Kenya; 24 Programme National de Lutte Contre l’Onchocercose et la Cécité et les Maladies Tropicales Négligées, Conakry, Guinea; 25 Ministry of Health, Rabat, Morocco; 26 Ministry of Health, Dar es Salaam, United Republic of Tanzania; 27 l'unité d'élimination du trachome, PNMTN, Ouagadougou, Burkina Faso; 28 Department of Ophthalmology, University of Malawi, College of Medicine, Blantyre, Malawi; 29 National Institute for Medical Research, Dar es Salaam, United Republic of Tanzania; 30 Federal Ministry of Health, Addis Ababa, Ethiopia; 31 Ministry of Health, Islamabad, Pakistan; 32 Ministry of Health, South Sudan; 33 Ministry of Health, Port Vila, Vanuatu; 34 Department of Epidemiology and Disease Control, Ministry of Health & Child Welfare, Harare, Zimbabwe; 35 Ministry of Health, Lilongwe, Malawi; 36 Ministerio da Saude, Department of Neglected Tropical Diseases, Maputo, Mozambique; 37 Department of Biochemistry, University of Zimbabwe, Harare, Zimbabwe; 38 Ministère de la Santé, Bissau, Guinea-Bissau; 39 Department of Medical Microbiology, College of Health Sciences, University of Zimbabwe, Harare, Zimbabwe; 40 Minister de la Sante, Nouakchott, Mauritania; 41 Nepal Netra Jyoti Sangh, Kathmandu, Nepal; 42 Sightsavers Nigeria, Kaduna, Nigeria & Department of Ophthalmology, Jos University, Jos, Nigeria; 43 RTI International, Dar es Salaam, United Republic of Tanzania; 44 Nigeria Federal Ministry of Health, Abuja, Nigeria; 45 RTI International, Washington DC, USA; 46 Christian Blind Mission, Bensheim, Germany; 47 Ministère de la santé et de l’Action Sociale, Dakar, Senegal; 48 Prevention of Blindness Programme, Ministry of Health, Phnom Penh, Cambodia; 49 Ministry of Health, Guatemala City, Guatemala; 50 Ministry of Health, Kathmandu, Nepal; 51 National Ophthalmology Centre, Vientiane, Lao People’s Democratic Republic; 52 National Program for the Prevention of Blindness, Ministry of Health, Vientiane, Lao People’s Democratic Republic; 53 Department of Information, Evidence and Research, World Health Organization, Geneva, Switzerland; 54 Department of Public Health, Ministry of Health, Asmara, Eritrea; 55 Ministère de la Santé, Bamako, Mali; 56 Trachoma Program, Ministry of Health, Kampala, Uganda; 57 Ministère de la Santé Publique, Bangui, Central African Republic; 58 Department of Ophthalmology, Peking Union Medical Colllege Hospital, Chinese Academy of Medical Sciences, Beijing, China; 59 Department of Control of Neglected Tropical Diseases, World Health Organization, Geneva, Switzerland; University of California Davis, UNITED STATES

## Abstract

**Background:**

Trichiasis is present when one or more eyelashes touches the eye. Uncorrected, it can cause blindness. Accurate estimates of numbers affected, and their geographical distribution, help guide resource allocation.

**Methods:**

We obtained district-level trichiasis prevalence estimates in adults for 44 endemic and previously-endemic countries. We used (1) the most recent data for a district, if more than one estimate was available; (2) age- and sex-standardized corrections of historic estimates, where raw data were available; (3) historic estimates adjusted using a mean adjustment factor for districts where raw data were unavailable; and (4) expert assessment of available data for districts for which no prevalence estimates were available.

**Findings:**

Internally age- and sex-standardized data represented 1,355 districts and contributed 662 thousand cases (95% confidence interval [CI] 324 thousand–1.1 million) to the global total. Age- and sex-standardized district-level prevalence estimates differed from raw estimates by a mean factor of 0.45 (range 0.03–2.28). Previously non- stratified estimates for 398 districts, adjusted by ×0.45, contributed a further 411 thousand cases (95% CI 283–557 thousand). Eight countries retained previous estimates, contributing 848 thousand cases (95% CI 225 thousand-1.7 million). New expert assessments in 14 countries contributed 862 thousand cases (95% CI 228 thousand–1.7 million). The global trichiasis burden in 2016 was 2.8 million cases (95% CI 1.1–5.2 million).

**Interpretation:**

The 2016 estimate is lower than previous estimates, probably due to more and better data; scale-up of trichiasis management services; and reductions in incidence due to lower active trachoma prevalence.

## Author summary

As an individual with trichiasis blinks, the eyelashes abrade the cornea, which can lead to corneal opacity and blindness. Through high quality surgery, which involves altering the position of the eyelid margin, it is possible to reduce the number of people with trichiasis. Accurate estimates of the number of persons with trichiasis and their geographical distribution are needed in order to effectively align resources for surgery and other necessary services. We obtained district-level trichiasis prevalence estimates for 44 endemic and previously-endemic countries. We used the most recently available data and expert assessments to estimate the global burden of trichiasis. We estimated that in 2016 the global burden was 2.8 million cases (95% CI 1.1–5.2 million).

The 2016 estimate is lower than previous estimates, probably due to more and better data; scale-up of trichiasis management services; and reductions in incidence due to lower active trachoma prevalence.

## Introduction

Trachoma, a neglected tropical disease, is endemic or has recently been endemic in more than 50 countries[1]. It affects the most impoverished people of the world. Improved living standards are credited for the disappearance of trachoma from Europe and North America, but in many less developed countries, trachoma is still a public health problem, and contributes to the continued suffering and deepening of poverty of millions of people.

*Chlamydia trachomatis* is the causative organism. Repeated ocular chlamydial infection results in chronic inflammation, characterised by sub-epithelial follicles in the tarsal conjunctiva, which may be sufficiently large and numerous to meet the definition of trachomatous inflammation—follicular (TF), a key sign for assessing trachoma prevalence[2]. Over time, with repeated reinfection, scarring may develop; this scarring can eventually cause the eyelid to turn inwards in some people, resulting in eyelashes touching the globe. This is called trachomatous trichiasis (TT) and is very painful[3]. As an individual with trichiasis blinks, the eyelashes abrade the cornea, which can lead to corneal opacity and blindness[4].

The World Health Organization (WHO) endorses population based prevalence surveys (PBPSs) to guide decisions regarding implementation of interventions for trachoma elimination[5]. The typical survey design involves two-stage cluster sampling, which uses probability-proportional-to-size methodologies to select 20–30 clusters. The outputs are estimates of the prevalence of TF in children aged 1–9 years, and the prevalence of TT in adults aged 15 years and older[6].

Through high quality surgery, which involves altering the position of the eyelid margin,[7] it is possible to reduce the number of people with TT. Accurate estimates of the number of persons with TT (the TT backlog) and their geographical distribution are needed in order to effectively align resources for surgery and other necessary services.

In 2009, Mariotti et al estimated the global TT backlog to be 8.2 million people[8]. This estimate was derived by summarizing a combination of published and unpublished information. First, a literature review was performed to identify published prevalence data. Where published data were not available, unpublished data were compiled from the Eleventh (2007) Meeting of the WHO Alliance for the Global Elimination of Trachoma by 2020[9]. Where information was still missing, unpublished reports were provided by health ministries. Finally, if none of these resources were available, data were extrapolated from a proxy country believed to have common epidemiological conditions and demographic structure.

There are many uncertainties inherent in the 2009 estimate[8]. First, where PBPS data were available, the results were not systematically stratified by age and sex. This is problematic because women and the elderly are more likely than men and younger adults, respectively, to (1) be at home at the time that a house-to-house survey team calls, and (2) have TT. Second, in countries for which data were available, survey coverage was generally far from complete. Though not an invariable rule, surveys to estimate the prevalence of neglected diseases have a tendency to be done first in areas of high prevalence; for the 2009 estimate, if any data were available for a particular country, the TT prevalence figure from those data was applied across the yet-to-be-mapped suspected-endemic population. Third, the use of proxy countries is extremely subjective.

In 2012, WHO collected and collated provisional 2011 country reports, and published an updated figure for the global TT backlog. The total given was 7.3 million people, but the methodologies used in each country to generate national backlog figures were not described[10]; they are likely to have been highly heterogeneous.

From December 2012 to January 2016, the Global Trachoma Mapping Project (GTMP) sought to systematically complete the global trachoma map using standardized techniques for both collecting and analysing survey data[11]. Eligibility[12] and protocols [11]for mapping were determined by expert working groups. GTMP measured trachoma prevalence using gold standard PBPSs conducted at district level. People of all ages living in selected households of 1,546 districts across 29 countries were examined for trachoma, resulting in the examination of 2.6 million people[13]. GTMP analyses included stratifying trichiasis prevalence estimates against national population pyramids in an attempt to partially account for demographic differences between those examined and the national averages.

As a result of the GTMP, there are now high quality PBPS data available for most suspected-endemic areas that were previously unsurveyed. The availability of these data has catalysed the current attempt to generate a new estimate of the global trichiasis backlog.

## Methods

We updated previous global estimates using the best available data, according to the following hierarchy, with all areas worldwide that have trichiasis prevalence estimates eligible for inclusion. First, where GTMP data[11] were available, we used the age- and sex- stratified survey-level trichiasis prevalence estimates for adults. In August 2014, GTMP added examination for the presence or absence of trachomatous scarring (TS)[2] for all eyes determined to have trichiasis; prior to this, and for all non-GTMP data, trichiasis prevalence estimates included trichiasis with and without TS. All analyses of trichiasis were undertaken among adults aged 15 years and older only.

Second, where PBPSs had been done without the support of the GTMP, we requested de-identified individual-level raw survey data from national health ministries, or appropriate government-designated agencies. Where those data were provided by 1 March 2016, we applied the same age- and sex- stratification as was used in the GTMP[11].

Third, where PBPSs had been done but raw data were not available, survey-level prevalence estimates (whether stratified by age and sex or not) were obtained from country programs. When data had not been stratified, estimates were multiplied by the mean adjustment factor for districts in which age- and sex- stratification was possible.

Fourth, if prevalence estimates were not available, we reviewed previous estimates through desk reviews and communications with country programs. If there was adequate evidence to revise the estimates, new estimates were used; otherwise, the 2009 estimates[8] were retained.

The population data used in these analyses were derived from the UN population division (UNdata)[14] and www.worldpop.org[15]. As trachoma is a disease primarily affecting rural populations[4], we used rural population estimates for this purpose. Rural population pyramids were obtained from UNdata. Microsoft Excel (2007) was used to organize the datasets into 5-year age bands stratified by sex for each country. The percentage of the population within each stratum was estimated from the bands. The district level populations were derived from www.worldpop.org raster files[15] using the zonal statistics tool in ArcGIS 10.3[16]. The raster files used here are a matrix of geolocated pixels where each pixel contains population values. The sum values of the pixels falling within each district boundary was taken as the district value. A sensitivity analysis was also performed, comparing the www.worldpop.org population estimates to district population estimates provided by national trachoma elimination programs. The mean ratio between the national program estimates and www.worldpop.org was 0·97. Because of this close correlation, and for the purposes of standardizing our methods, the www.worldpop.org data were used throughout this analysis.

For population-based prevalence surveys done without GTMP support but for which raw data were available, the statistical software package R[17] was used to perform age- and sex- post-stratification. First, the crude prevalence was calculated for each cluster. Second, the prevalence was stratified for each cluster by weighting the proportion of each sex-specific five-year age band observed to have trichiasis by the proportion of the adults aged 15 years and older expected to have that age and sex in that district, if available, or (if not available), nation-wide. Third, the un-weighted arithmetic mean of the stratified cluster-level trichiasis proportions was taken as the district-level prevalence. Sampling weights were not needed here as the surveys used probability-proportional-to-size sampling and are therefore self-weighted. Once the initial post-stratification was completed, bootstrapping was undertaken on the dataset for each individual district to derive 95% confidence intervals (CIs). For a district surveyed by examining individuals in *n* clusters, this involved bootstrap resampling (with replacement) of *n* clusters, over 10,000 replications. The R code is provided in the Appendix.

Where PBPSs had been done but raw data were not available, CIs were constructed around the original estimate by applying an approximation to the binomial distribution $CI=p-\left(\overset{'}{z}\times s.e.\right)to\;p+\left(\overset{'}{z}\times s.e.\right)$, where p is the proportion of positive cases in the sample, s.e. is $\sqrt{p\left(1-p\right)/n}$, and $\overset{'}{z}$ is the standard normal distribution (95% CI = 1.96). Where the sample size was not available, a sample of 1000 was assumed. This method was used at the district-level when data were available and country-level when previous estimates were retained. When the district-level prevalence estimate was multiplied by the mean adjustment factor for districts in which age- and sex- stratification was possible, the upper and lower bounds of the corresponding confidence intervals were adjusted in the same way.

Post-stratified prevalence estimates and the lower and upper 95% CI bounds were multiplied by the 15-years-and-older population for the relevant district (projected to 2016) to provide an estimate of the number of persons with trichiasis.

Health ministries of endemic countries reviewed the output; for three countries, as will be noted in the results section, the estimate was subsequently reduced to account for implementation of trichiasis surgery programs since the time of survey. This changed the category of estimates in the countries involved from estimates based on PBPS data to “expert assessments”.

For expert backlog assessments, confidence intervals were constructed using the relative precisions of all datasets for which bootstrapped CIs could be determined. The range of relative upper bound and lower bound precisions were trimmed to exclude 10% of the greatest and least values. The means of the remainder were applied.

Upper (and lower) CI bounds around district-level estimates, regardless of the method used for calculation, were summed to generate country-level, region-level and global estimates of the upper (and lower) CI bounds.

A sensitivity analysis was performed where communications with country programs estimated a value of zero. This involved selecting proxy countries based on similar geography and hygiene and sanitation situations and calculating the ratio of estimated country backlog over country population. This ratio was then applied to the zero value countries to estimate a backlog. Hygiene and sanitation data were derived from the WHO/UNICEF Joint Monitoring Program for Water Supply, Sanitation and Hygiene (JMP) [18].

The study was approved by the Research Ethics Committee of the London School of Hygiene & Tropical Medicine (11208). The corresponding author had full access to all the data and had final responsibility for the decision to submit for publication.

## Results

**Fig 1:**
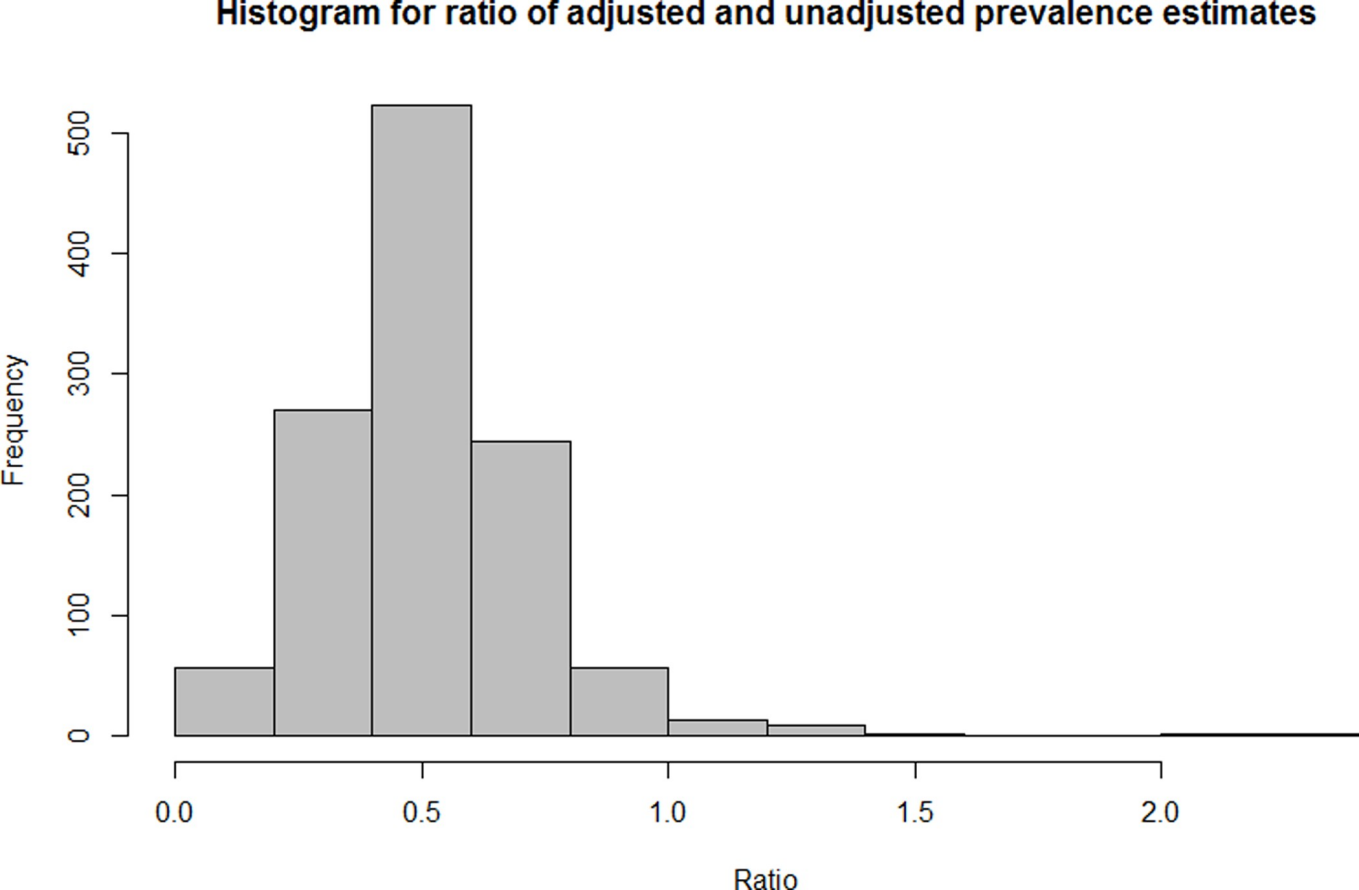
Histogram for ratio of adjusted and unadjusted prevalence estimates.

The 2009 estimate included 57 historically trachoma-endemic countries; this updated estimate reviewed 65 known and suspected trachoma-endemic countries. Descriptive statistics for cluster level data are found in the individual country-level publications. Data from 1,355 districts in 31 countries were age- and sex- stratified and contribute an estimated 662 thousand cases (95% confidence interval (CI) 324 thousand-1.1 million) to the global total. These data were made up of 29,847 clusters, averaging 22 clusters per district. Adjusting prevalence estimates by age and sex reduced raw district-level estimates by a mean factor of 0.45 (range 0.03–2.28; Fig 1). This value was calculated by averaging the ratio of adjusted and unadjusted prevalence estimates across districts that had raw data available. While the district-level estimates reduce by a mean factor of 0.45, the overall country-level backlog reduced by a mean factor of 0.56 (range 0.22–0.94) (Table 1).

**Table 1:** Estimated national-level trichiasis backlogs, 2016, with comparisons to the corresponding estimates for 2009.

WHO region	Country	2009 estimate[8] (data generally from 2007)	Source for 2016 estimate (1)	2016 estimate [year of data collection] (2) (95% confidence intervals)	Representativeness
African Region	Algeria	86,700	Retained previous estimate	86,700 (23,022–175,625)	
	Angola	no data	Expert assessment (3)	0	
	Benin	7,600	GTMP[20]	11,782 [2014–2015] (5,148–20,861)	Estimate based on 12 age- and sex-adjusted PBPS datasets covering all districts in the country where evidence indicates trachoma may be a public health problem
	Botswana	32,900	Expert assessment (3)	0	
	Burkina Faso	32,800	PBPS–no raw data available	19,443 [2005–2015] (5,381–35,236)	Estimate based on 63 PBPS datasets covering all districts in the country
	Burundi	no data	Expert assessment (3)	0	
	Cameroon	47,200	PBPS–no raw data available	698 [2012–2014] (27–1,523)	Estimate based on 1) 35 age- and sex-adjusted PBPS datasets, plus 2) 15 PBPS datasets, covering all districts in the country where evidence indicates trachoma may be a public health problem
			PBPS–raw data available	10,743 [2010–2014] (5,705–16,525)	
	Central African Republic	1,000	PBPS–no raw data available	6,539 [2011] (1,991–11,259)	Estimate based on 8 PBPS datasets; security concerns prevented the remaining 7 districts in the country where evidence indicates trachoma may be a public health problem from being surveyed
	Chad	34,300	GTMP	24,597 [2014–2015] (8,513–44,924)	Estimate based on 1) 43 age- and sex-adjusted PBPS datasets, plus 2) 14 PBPS datasets. At the time of publication 1 district where evidence indicates trachoma may be a public health problem remained to be surveyed
			PBPS–no raw data available	23,024 [2002] (15,365–30,683)	
	Congo		GTMP	0 [2015]	Estimate based on 1 age- and sex-adjusted PBPS dataset covering all districts in the country where evidence indicated that trachoma might have been a public health problem
	Côte d'Ivoire	59,900	GTMP	1, 216 [2015] (176–2,582)	Estimate based on 10 age- and sex-adjusted PBPS datasets covering all districts in the country where evidence indicates trachoma may be a public health problem
	Democratic Republic of the Congo	no data	GTMP[21]	33,333 [2014–2016] (18,369–52,870)	Estimate based on 30 age- and sex-adjusted PBPS datasets; security concerns prevented 1 district from being surveyed. At the time of publication an additional 9 districts where evidence indicates trachoma may be a public health problem remained to be surveyed.
	Eritrea	42,000	GTMP	774 [2014] (274–1,308)	Estimate based on 1) 2 age- and sex-adjusted PBPS datasets, plus 2) 36 PBPS datasets; security concerns prevented the remaining 1 district in the country where evidence indicates trachoma may be a public health problem from being surveyed
			PBPS–no raw data available	24,793 [2006–2014] (15,005–34,823)	
	Ethiopia	1,272,600	Expert assessment[22–26] (4)	693,037 [2012–2016] (184,025–1,403,858)	Expert assessment based on 1) a backlog calculated from 196 age- and sex-adjusted PBPS datasets; security concerns prevented the remaining 16 districts in the country where evidence indicates trachoma may be a public health problem from being surveyed; minus 2) programmatic TT surgery output from 2012–2016
	Gambia	10,500	PBPS–no raw data available	0 [2016]	PBPS datasets covering all districts in the country where evidence indicates trachoma may be a public health problem
	Ghana	3,000	PBPS–no raw data available	1,379 [2007–2008] (188–2,875)	Estimate based on 23 PBPS datasets, covering all districts in the country where evidence indicated that trachoma may have been a public health problem
	Guinea	25,100	GTMP	5,523 [2014–2016] (1,141–11,834)	Estimate based on 31 age- and sex-adjusted PBPS datasets, covering all districts in the country where evidence indicates trachoma may be a public health problem
			PBPS–raw data available	24,302 [2011–2013] (13,035–38,193)	
	Guinea-Bissau	16,400	PBPS–raw data available	21,255 [2005] (12,975–29,638)	Estimate based on 11 PBPS datasets, covering all districts in the country where evidence indicates trachoma may be a public health problem
	Kenya	306,800	PBPS–no raw data available	30,195 [2004–2012] (21,404–39,116)	Estimate based on 1) 3 age- and sex-adjusted PBPS datasets, plus 2) 12 PBPS datasets, covering all districts in the country where evidence indicates trachoma may be a public health problem
			PBPS–raw data available	21,363 [2004] (13,583–31,471)	
	Malawi	33,400	GTMP[27, 28]	13,446 [2013–2015] (3,302–27,937)	Estimate based on 1) 30 age- and sex-adjusted PBPS datasets, plus 2) 3 PBPS datasets, covering all districts in the country where evidence indicates trachoma may be a public health problem
			PBPS–no raw data available	1,128 [2012] (227–2,124)	
			PBPS–raw data available	817 [2014] (48–1,918)	
	Mali	67,600	Expert assessment (5)	13,852 (3,678–28,059)	Estimate based on 1) 55 PBPS datasets, covering all districts in the country minus 2) programmatic TT surgery output between survey completion and 2016
	Mauritania	2,500	PBPS–no raw data available	1,556 [2004–2013] (250–3,325)	Estimate based on 29 PBPS datasets, covering all districts in the country where evidence indicates trachoma may be a public health problem
	Mozambique	60,500	GTMP[29]	18,817 [2013–2014] (3,958–39,883)	Estimate based on 1) 99 age- and sex-adjusted PBPS datasets, covering all districts in the country where evidence indicates trachoma may be a public health problem
			PBPS–raw data available	96 [2015] (89–111)	
	Namibia	6,100	Expert assessment (3)	0	
	Niger	59,600	Expert assessment (8)	40,592 (9,712–83,784)	Estimate based on 1) 49 PBPS datasets, covering all districts in the country where evidence indicates trachoma may be a public health problem minus 2) programmatic TT surgery output between survey completion and 2016
	Nigeria	627,300	GTMP[30–34]	193,951 [2013–2014] (111,251–297,277)	Estimate based on 1) 294 age- and sex-adjusted PBPS datasets, plus 2) 103 PBPS datasets, security concerns prevented the remaining 47 districts in the country where evidence indicates trachoma may be a public health problem from being surveyed
			PBPS–no raw data available	90,865 [2003–2014] (56,635–126,704)	
			PBPS–raw data available	3,800 [2014–2015] (1,801–6,295)	
	Senegal	129,800	GTMP	12,707 [2014] (6,823–19,364)	Estimate based on 1) 20 age- and sex-adjusted PBPS datasets, plus 2) 27 PBPS datasets, covering all districts in the country where evidence indicates trachoma may be a public health problem
			PBPS–no raw data available	30,905 [2000–2012] (18,450–43,437)	
			PBPS–raw data available	3,077 [2015] (1,502–4,695)	
	South Sudan	no data	Expert assessment	81,093 (21,533–164,267)	Estimate provided by the Ministry of Health, South Sudan. To date, only a quarter of South Sudan has been mapped–this figure is likely to be an under-estimate for the country
	Togo	2,900	PBPS–no raw data available	318 [2009] (45–680)	Estimate based on 3 PBPS datasets covering all districts in the country where evidence indicates trachoma may be a public health problem
	Uganda	610,600	GTMP	680 [2014] (253–1,200)	Estimate based on 1) 39 age- and sex-adjusted PBPS datasets, plus 2) 5 PBPS datasets covering all districts in the country where evidence indicates trachoma may be a public health problem
			PBPS–no raw data available	14,559 [2008–2012] (9,635–19,483)	
			PBPS–raw data available	65,329 [2013–2016] (39,924–149,042)	
	United Republic of Tanzania	214,800	GTMP[35, 36]	8,096 [2014] (2,491–15,225)	Estimate based on 97 age- and sex-adjusted PBPS datasets, covering all districts in the country where evidence indicates trachoma may be a publi c health problem
			PBPS–raw data available	63,157 [2004–2016] (28,844–87,028)	
	Zambia	8,500	GTMP	1,524 [2015] (712–2,670)	Estimate based on 1) 7 age- and sex- adjusted PBPS datasets, plus 2) 3 PBPS datasets covering all districts in the country where evidence indicates trachoma may be a public health problem
			PBPS–no raw data available	2,168 [2010–2015] (1,289–3,047)	
	Zimbabwe	44,100	GTMP	6,765 [2014–2015] (3,133–10,988)	Estimate based on 16 age- and sex-adjusted PBPS datasets covering all districts in the country where evidence indicates trachoma may be a public health problem
Eastern Mediterranean Region	Afghanistan	83,100	Retained previous estimate	83,100 (22,066–168,332)	
	Djibouti	3,900	Expert assessment (6)	75 (20–152)	
	Egypt	35,400	GTMP	35,362 [2015] (16,003–58,414)	Estimate based on 1) 4 age- and sex-adjusted PBPS datasets, plus 2) 1 PBPS dataset
			PBPS–no raw data available	35,400 [2015] (30,596–40,204)	
	Iran (Islamic Republic of)	49,300	Expert assessment (3)	0	
	Iraq	43,900	Expert assessment (3)	0	
	Libya	13,200	Expert assessment (7)	33,400 (8,869–67,657)	
	Morocco	6,400	PBPS–no raw data available	0 [2015]	Estimate based on PBPS datasets covering all districts in the country where evidence indicated that trachoma may have been a public health problem
	Oman	600	PBPS–no raw data available	600 [2005] (41–619)	Estimate based on PBPS datasets covering all districts in the country where evidence indicated that trachoma may have been a public health problem
	Pakistan	71,700	GTMP	5,330 [2015] (1,160–10,732)	Estimate based on 1) 42 age- and sex-adjusted PBPS datasets, plus 2) 31 PBPS datasets; security concerns prevented 15 districts from being surveyed. At the time of publication an additional 1 district where evidence indicates trachoma may be a public health problem remained to be surveyed.
			PBPS–no raw data available	23,420 [2012] (12,205–34,975)	
	Somalia	10,300	Retained previous estimate	10,300 (2,735–20,864)	
	Sudan	528,100	GTMP[37]	22,508 [2014–2015] (14,208–33,449)	Estimate based on 1) 109 age- and sex-adjusted PBPS datasets, plus 2) 22 PBPS datasets; security concerns prevented 12 districts from being surveyed. At the time of publication an additional 1 district where evidence indicates trachoma may be a public health problem remained to be surveyed.
			PBPS–no raw data available	6,751 [2006–2013] (2,599–10,903)	
			PBPS–raw data available	36,982 [2007–2010] (7,070–80,470)	
	Yemen	270,800	GTMP	5,821 [2013–2015] (896–13,165)	Estimate based on 1) 42 age- and sex-adjusted PBPS datasets; security concerns prevented the remaining 38 districts in the country where evidence indicates trachoma may be a public health problem from being surveyed
Region of the Americas	Brazil	58,000	Retained previous estimate	58,000 [2003–2006] (15,401–117,488)	Estimate based on PBPS datasets covering all districts in the country where evidence indicates trachoma may be a public health problem
	Colombia	no data	GTMP	48 [2015] (0–123)	Estimate based on 1) 3 age- and sex-adjusted PBPS datasets, plus 2) 6 PBPS datasets. At the time of publication an additional 3 districts where evidence indicates trachoma may be a public health problem remained to be surveyed.
			PBPS–no raw data available	23 [2003–2009] (4–44)	
	Guatemala	30	PBPS–no raw data available	543 [2011] (268–819)	Estimate based on 2 PBPS datasets covering all districts in the country where evidence indicates trachoma may be a public health problem
	Mexico	20	Expert assessment (3)	0	
South-East Asia Region	India	443,000	Retained previous estimate	443,000 (117,632–897,368)	
	Nepal	138,800	PBPS–no raw data available	25,240 [2001–2012] (16,889–33,631)	Estimate based on 1) 30 age- and sex-adjusted PBPS datasets, plus 2) 17 PBPS datasets covering all districts in the country where evidence indicates trachoma may be a public health problem
			PBPS–raw data available	3,252 [2002–2009] (462–7,323)	
Western Pacific Region	Australia	1,100	Retained previous estimate	1,100 (292,2,228)	
	Cambodia	29,200	GTMP[38]	4,999 [2014–2015] (729–10,676)	Estimate based on 14 age- and sex-adjusted PBPS datasets covering all districts in the country where evidence indicates trachoma may be a public health problem
	China	2,330,600	PBPS–no raw data available	71,280 (70,399–72,161)	Estimate based on PBPS datasets covering all districts in the country where evidence indicated that trachoma may have been a public health problem before 2012
	Fiji	800	PBPS using the methods of the GTMP[39] and subsequent investigation[40]	0 [2013]	Estimate based on 1 age- and sex-adjusted PBPS dataset covering all districts in the country where evidence indicates trachoma may be a public health problem
	Kiribati	100	GTMP	69 [2015] (30–108)	Estimate based on 1 age- and sex-adjusted PBPS dataset covering all districts in the country where evidence indicates trachoma may be a public health problem
	Lao People’s Democratic Republic	900	GTMP[41]	630 [2013–2015] (0–1,728)	Estimate based on 16 age- and sex-adjusted PBPS datasets covering all districts in the country where evidence indicates trachoma may be a public health problem
	Myanmar	65,800	Retained previous estimate	65,800 (17,472–133,289)	
	Nauru	0	Expert assessment (3)	0	
	Papua New Guinea	5,800	GTMP[42]	156 [2015] (0–362)	Estimate based on 7 age- and sex-adjusted PBPS datasets covering all districts in the country where evidence indicates trachoma may be a public health problem
	Solomon Islands	500	GTMP[43]	44 [2013] (4–104)	Estimate based on 1) 3 age- and sex-adjusted PBPS datasets, plus 2) 5 PBPS datasets, covering all districts in the country where evidence indicates trachoma may be a public health problem
			PBPS–no raw data available	8 [2012] (0–23)	
	Vanuatu	200	GTMP[44]	48 [2014] (47–50)	Estimate based on 1 age- and sex-adjusted PBPS datasets covering all districts in the country where evidence indicates trachoma may be a public health problem
	Viet Nam	210,000	Expert assessment (6)	100,000[19] (26,553–202,566)	

1) Unless otherwise specified, “Retained previous estimate” refers to the 2009 estimate by Mariotti et al[8]

2) Individuals examined in these surveys were men and women aged ≥15 years

3) Health ministry reports that there is no evidence of trichiasis being a public health problem, or that evidence indicates that trichiasis is not a public health problem

4) Estimate provided by the Federal Ministry of Health of Ethiopia; determined by calculating the backlog indicated by the most recent population-based prevalence survey in each trachoma-endemic district and subtracting from it the number of individuals with trichiasis managed by the health system since those surveys; 95% CIs were calculated using the bootstrapping approach described in the methods and provided by the Ministry

5) Estimate provided by the Ministère de la Santé, Mali; determined by calculating the backlog indicated by the most recent population-based prevalence survey in each trachoma-endemic district and subtracting from it the number of individuals with trichiasis managed by the health system since those surveys; 95% CIs were calculated using the bootstrapping approach described in the methods and provided by the Ministry

6) derived from information provided at the 2010 WHO Alliance for GET2020 meeting

7) Estimate informed by data from a Rapid Assessment of Avoidable Blindness (RAAB) survey

Estimates from PBPSs in 398 districts could not be stratified by age and sex because the original datasets were unavailable; the unadjusted backlogs in these districts totalled 781 thousand cases. We adjusted these estimates by multiplying them across the board by 0.45, yielding 411 thousand cases (95% CI 283–557 thousand).

Based on new expert assessment of available data, it was determined that eight countries no longer have a trichiasis backlog. These countries were Angola, Botswana, Burundi, Iran, Iraq, Namibia, Nauru, and Mexico. For six countries—Djibouti, Ethiopia, Libya, Mali, Niger and South Sudan—local expert assessment generated revised, non-zero estimates. In Ethiopia, Niger and Mali, the local expert assessment was undertaken at the request of the health ministry, where, for each district the number of surgeries done since the most recent prevalence survey (as reported annually to WHO) was subtracted from the estimated district-level backlog. (Incorporating surgical delivery in these three countries reduced the global backlog estimate by 124,433 cases: Ethiopia 69,133, Mali 32,083, and Niger 23,217.) For one country, other published estimates[19] were used.

**Fig 2:**
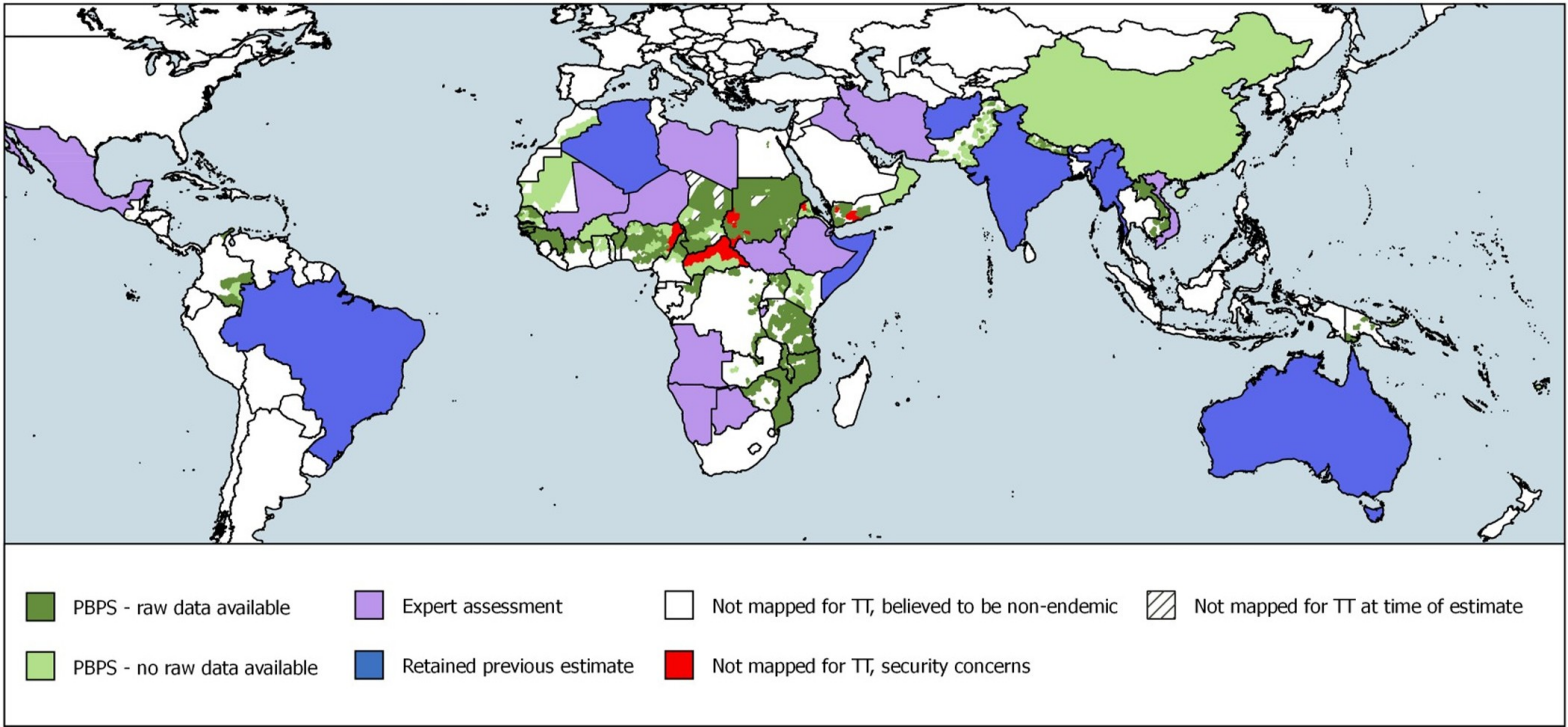
Distribution of analysis methodology.

**Fig 3:**
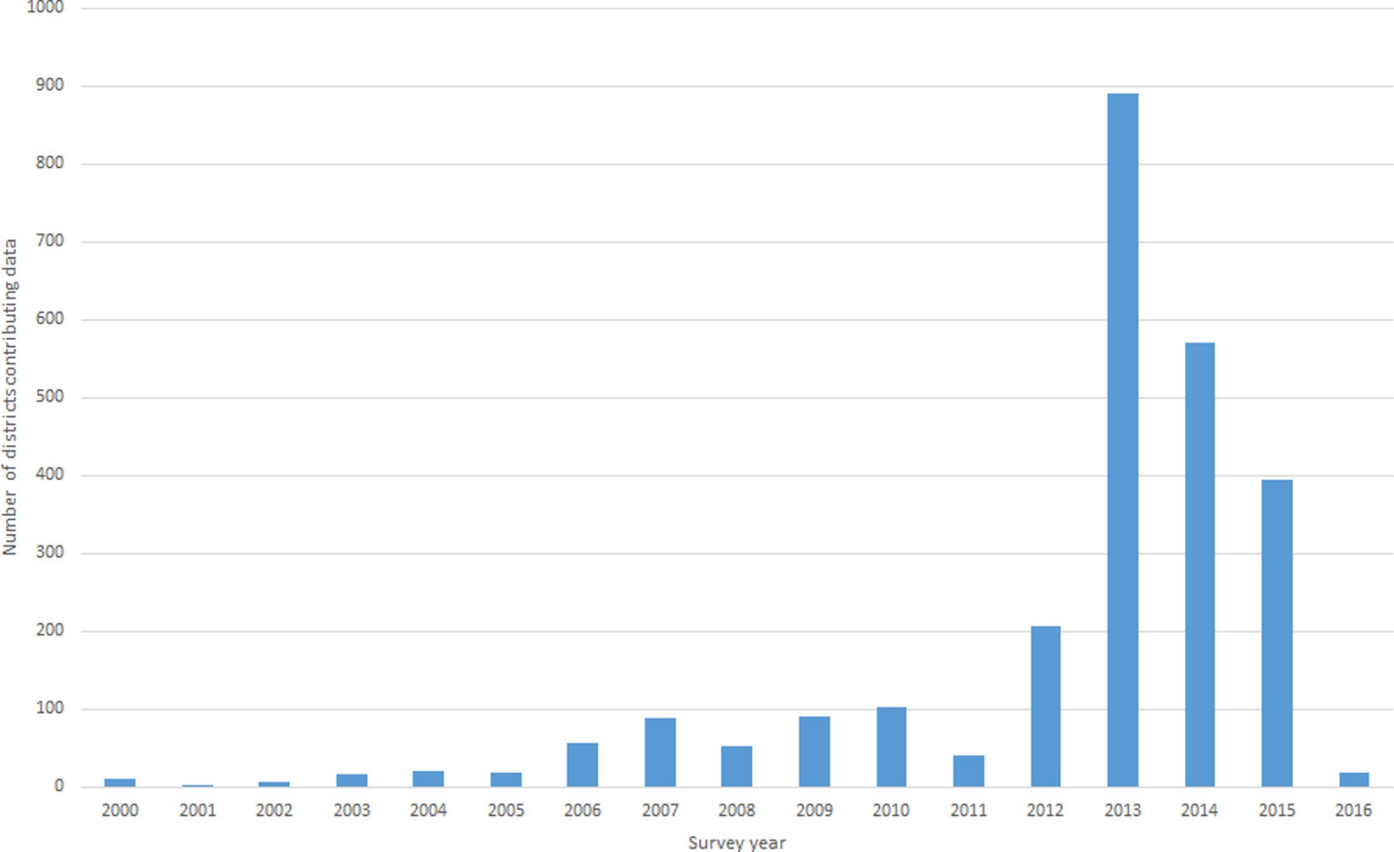
Number of districts contributing data to the overall estimate, by year of survey.

Finally, there were seven countries for which the 2009 estimates were retained. The country-level data derived from previous estimates and expert assessments contributes 61.4% of the overall estimate (Table 2 and Fig 2). Data included in the overall estimate had been collected from 2000–2016 (Fig 3).

**Table 2:** Backlog estimate attributed to each analysis methodology.

Method	Geography	Backlog estimate	Percent of overall estimate
PBPS–raw data available	1,355 districts	662,403	23.8%
PBPS–no raw data available	398 districts	410,835	14.8%
Retained previous estimate	8 countries	848,000	30.5%
Expert assessment	14 countries	862,049	31.0%

Through the inclusion of GTMP data, adjusting older datasets by age and sex, and obtaining current local expert assessment of available data, the global backlog estimate reduces from 2009’s 8.2 million[8] (or 2012’s 7.3 million[10]) to 2.8 million (95% CI 1.1–5.2 million) in 2016 (Tables 2 and 3). The updated estimate reduced to zero cases in twelve countries, namely: Angola, Botswana, Burundi, Congo, Fiji, Gambia, Iraq, Islamic Republic of Iran, Morocco, Mexico, Namibia, and Nauru. (Updated survey data demonstrated zero prevalence in Congo, Gambia, Morocco and Fiji, while health ministries in Angola, Botswana, Burundi, Iraq, Islamic Republic of Iran, Mexico, Namibia and Nauru reported no current evidence of trichiasis.) The sensitivity analysis performed for countries where the health ministry reported no current evidence of trichiasis suggests these zero estimates may contribute to a global underestimate by a margin of 13,285, a small difference in the context of the overall estimate, particularly given uncertainty from other sources. The outputs from this analysis are provided in the Appendix.

**Table 3:** Estimated region-level trichiasis backlogs, 2016, with comparisons to the corresponding estimates for 2009.

WHO Region	2009 Estimate	2016 Estimate				Ratio (2016 estimate / 2009 estimate)
		Retained previous estimate or expert assessment (95% CI)	Not post-stratified (reduced by a factor of 0·45) (95% CI)	Post-stratified (95% CI)	Total (95% CI)	
African Region	3,846,500	915,274 (241,969–1,855,593)	247,570 (150,310–363,186)	547,151 (283,052–913,841)	1,709,995 (675,331–3,132,620)	0.45
Region of the Americas	58,050	58,000 (15,401–117,488)	566 (272–863)	48 (0–123)	58,614 (15,673–118475)	1.01
Eastern Mediterranean Region	660,000	126,875 (33,690–257,006)	66,171 (45,441–86,702)	106,003 (39,337–196,230)	299,049 (118,467–539,937)	0.52
South-East Asia Region	485,000	443,000 (117,632–897,368)	25,240 (16,889–33,631)	3,252 (462–7,323)	471,492 (134,982–938,323)	0.97
Western Pacific Region	2,630,000	166,900 (44,318–338,083)	71,288 (70,399–72,184)	5,947 (810–13,027)	244,135 (115,527–423,294)	0.09
Total	8,248,050	1,710,049 (453,010–3,465,538)	410,835 (283,311–556,567)	662,403 (323,661–1,130,543)	2,783,285 (1,059,980–5,152,649)	0.35

In our estimates, data collected in surveys set up prior to August 2014 include all trichiasis, irrespective of the presence or absence of trachomatous conjunctival scarring. Data collected in surveys set up after August 2014 contributed data on trichiasis in eyes that also demonstrated trachomatous conjunctival scarring (or had an eyelid that could not be everted, due to presumed dense scar), thereby including only those cases of trichiasis attributable to trachoma. In both scenarios the data represent both “managed” and “unmanaged” trichiasis, irrespective of whether individuals have previously been offered corrective surgery or epilation[45].

## Discussion

Trichiasis remains a significant public health problem in many countries, with a global backlog estimated for 2016 at 2.8 million people, 61% of whom live in sub-Saharan Africa. While there were methodological challenges in generating the 2009 estimate, it was the best estimate that could be made with the information then available. The considerable reduction from that estimate to the one generated here is likely to be the result of a combination of factors. First, there are now more and better data derived from rigorous surveys. Second, in many countries, there has been impressive programmatic scale up to manage TT, conducted by complex networks of governments and their partners. Third, there is likely to be an effect on the incidence of TT from the intensive efforts to reduce active trachoma prevalence in many contexts; such efforts have been ramping up in endemic countries since the World Health Assembly’s 1998 commitment to global elimination of trachoma[46]. Teasing out the relative contribution of each of these factors is not possible at the present time, but regardless of cause, the reduction is welcome news for global health.

A better understanding of the backlog and distribution of trichiasis cases is necessary to effectively plan for surgical services and other components of management of individuals with trichiasis. To reduce TT prevalence in each district of each endemic country to <0.2% in adults, which is the defined elimination threshold for TT[47], at least 2.0 million people will need to have their TT appropriately managed. From available data, it is estimated that 56% of people with trichiasis have bilateral trichiasis.

While our calculations lessen the uncertainty around the global backlog estimate, important limitations remain. Expert assessment of available data was used for 14 endemic countries, in eight of which the estimate was zero cases. The sensitivity analysis performed on these eight countries suggests that the zero estimates could contribute to global underestimation by a margin of 13,285. Ethiopia, Mali, and Niger have undergone intensive surgical scale-up in the time since the most recent round of prevalence surveys. Because of this programmatic output, and a lack of consensus around how to counterbalance backlog reduction with new incident cases and post-surgical recurrence (both of which are inescapable, but presently difficult to accurately quantify) these countries provided results based on expert assessment—albeit assessments grounded on 196 (Ethiopia), 55 (Mali) and 49 (Niger) PBPS datasets, and corresponding district-level data on surgical output. Uncertainty is greatest among the eight countries in which previous estimates were retained; these countries accounted for 848 thousand cases of trichiasis (31% of the global estimate) and further investigation is needed here. India, which accounts for 443,000 cases of trichiasis (52% of the total retained estimate) is a particular priority. A second ongoing uncertainty stems from the unavailability of raw data for 398 districts, for which, as a result, age- and sex- stratification was not possible. As noted in our calculations, there is a mean prevalence reduction against the raw prevalence estimate of ×0·45, which was applied to previously-unstratified district-level estimates. Third, our CIs reflect statistical uncertainty. Because statistical uncertainty is uncorrelated across countries, our method of aggregating CIs across administrative boundaries overestimates statistical uncertainty at regional and global levels. However, there are other sources of uncertainty in addition to statistical uncertainty, so it is not possible to say whether our regional and global CIs are biased. Fourth, we have implicitly assumed the prevalence of TT in those aged <15 years to be negligible. This is generally valid, but was a necessary assumption because of the unavailability of data on paediatric trichiasis in many pre-GTMP surveys. Fifth, it is possible that there are some population-based data on trichiasis of which we are unaware, but the partnerships engendered within the WHO Alliance for GET2020 [48]and the existence of the Trachoma Atlas (www.trachomaatlas.org) seem to us to make it unlikely that there are many estimates in this category. Finally, some of the surveys contributing data were conducted more than a decade ago (Fig 2). Although we note above that reductions in active trachoma prevalence are likely to have reduced the incidence of TT over time, we cannot adjust for such reductions within our data. Similarly, we are unable to adjust for the effects of all-cause mortality in patients with TT, population ageing, and other broad demographic changes that have occurred since raw data were collected.

Ongoing lack of trichiasis (and TF) prevalence data for 235 suspected-endemic districts is due to local insecurity, and the global trachoma community stands ready to support national governments to undertake the needed mapping in those populations, when and if security conditions improve to allow safe conduct of fieldwork.

It is recognized[11] that the PBPS methodology described earlier is potentially imprecise in estimating TT against the WHO elimination target of ≤0.2% in adults. Thus, at district level, the uncertainty around the estimates of the TT backlog can be large. This is reflected in our CIs. Work is underway to try to develop more reliable methodologies for estimating the prevalence of trichiasis.

At present, the diagnosis of TT is based on the presence of clinical trichiasis (one or more lashes touching the globe or evidence of recent epilation of in-turned eyelashes) plus residence in a (presumed) trachoma-endemic setting. This definition is by nature somewhat circular and may need review, as it inevitably leads to classification of trichiasis as TT regardless of whether trachomatous conjunctival scarring is present or not. Trichiasis without trachomatous conjunctival scarring may be age-related or due to trauma, distichiasis, epiblepharon or other inflammatory disease[49]; the sight-threatening potential and optimal management strategies for non-trachomatous trichiasis still require further investigation[50].

Assumptions were made when adjusting the data for age and sex. First, we assumed that ages reported in surveys were accurate. However, it is reasonable to hypothesise that during a survey, individuals demonstrate a terminal digit preference when stating their age. Second, this analysis assumed that UNdata population pyramids used were representative of all districts for which survey data were available. Finally, as already noted, we assumed zero trichiasis cases in the population aged 14 years and younger; studies demonstrate a high correlation between trichiasis and increased age[51–54].

These calculations of national and global TT burdens are point-prevalence estimates based on data of differing vintages; they will change as additional surveys, baseline or impact, are undertaken. Other than for Ethiopia, Mali and Niger, the estimates do not take into account the number of surgeries done during the period between the most recent survey and the cut-off date for this analysis. Nevertheless, estimates of current national TT backlog are essential for countries to appropriately allocate resources for surgical campaigns. Future district-level impact surveys in countries with trachoma elimination programs will provide progressively improved evidence for countries to assess their residual TT burden and, ultimately, validate elimination of trichiasis as a public health problem. Baseline surveys of trachoma are still needed in a handful of countries (e.g., Egypt, Somalia and Central African Republic); these will lead to revisions in national estimates. In some settings, there will be a need to undertake trichiasis-only surveys [55] in order to assess progress to elimination. Unfortunately, insecurity may continue to limit surveys in a few, but not all, of the settings in which previous estimates were retained. Where possible, surveys should be undertaken. Lack of primary data will hamper progress to the declaration of global elimination of trachoma as a public health problem. As new primary survey findings become available it will be possible to update global figures. Modelling may also be an important tool for addressing this challenge. Continued efforts by national health ministries, WHO, program partners and researchers to carry out baseline, impact, and pre-surveillance surveys drive revisions to the global elimination of trachomatous trichiasis as a public health problem.

## Supporting information

S1 Appendix R script for calculating TT prevalence normalized by age and sex. (DOCX)

S2 Appendix TT backlog sensitivity analysis. (DOCX)

S1 Checklist STROBE checklist. (DOCX)
