# Supplementary material for: The global burden of trichiasis in 2016
Source: PLoS Negl Trop Dis. 2019 Nov 25;13(11):e0007835. doi: 10.1371/journal.pntd.0007835 (PMC6901231; doi:10.1371/journal.pntd.0007835)
Supplement: S1 Appendix — (DOCX) [file pntd.0007835.s001.docx]

##### R script for calculating TT prevalence normalized by age and sex

| *##This script was modified from GTMP script written by Brian Chu and Rebecca Willis*  clean <- as.data.frame(read.csv("ISO_raw.csv"))  population<- read.csv("ISO_population.csv")  clean["tt_old"] <- 0  clean$tt_old[clean$TT=="1"] <- 1  *##generate age groups for the analysis, update for data available for specific country*  library(sqldf)  mydb_tt <- sqldf('SELECT EU, CLUSTER, (CASE  WHEN AGE BETIEN 15 AND 19 THEN "15_19"  WHEN AGE BETIEN 20 AND 24 THEN "20_24"  WHEN AGE BETIEN 25 AND 29 THEN "25_29"  WHEN AGE BETIEN 30 AND 34 THEN "30_34"  WHEN AGE BETIEN 35 AND 39 THEN "35_39"  WHEN AGE BETIEN 40 AND 44 THEN "40_44"  WHEN AGE BETIEN 45 AND 49 THEN "45_49"  WHEN AGE BETIEN 50 AND 54 THEN "50_54"  WHEN AGE BETIEN 55 AND 59 THEN "55_59"  WHEN AGE BETIEN 60 AND 64 THEN "60_64"  WHEN AGE BETIEN 65 AND 69 THEN "65_69"  WHEN AGE BETIEN 70 AND 74 THEN "70_74"  WHEN AGE BETIEN 75 AND 79 THEN "75_79"  WHEN AGE >= 80 THEN "80+"  END) AS AGE_GROUP, SEX,  COUNT(*) AS RESIDENTS,  SUM(CASE WHEN tt_old=1 THEN 1 ELSE 0 END) AS tt  FROM clean  WHERE AGE >= 15  GROUP BY EU, CLUSTER, AGE_GROUP, SEX  ORDER BY EU, CLUSTER, AGE_GROUP ASC, SEX DESC')  *##calculate unadjusted and adjusted TT prevalence*  ttprev_male <- as.data.frame(sqldf('SELECT mydb_tt.EU, mydb_tt.CLUSTER, mydb_tt.AGE_GROUP, mydb_tt.SEX, mydb_tt.RESIDENTS, mydb_tt.tt, population.percent_age_male AS age_Iight  FROM mydb_tt  LEFT JOIN population  ON mydb_tt.AGE_GROUP = population.age_group  WHERE mydb_tt.SEX = "1"'))  ttprev_female <- as.data.frame(sqldf('SELECT mydb_tt.EU, mydb_tt.CLUSTER, mydb_tt.AGE_GROUP, mydb_tt.SEX, mydb_tt.RESIDENTS, mydb_tt.tt, population.percent_age_female AS age_Iight  FROM mydb_tt  LEFT JOIN population  ON mydb_tt.AGE_GROUP = population.age_group  WHERE mydb_tt.SEX = "2"'))  *#append the dataframes*  ttprev <- as.data.frame(rbind(ttprev_male, ttprev_female))  #This creates the new column named "prev_unadj" filled with zeros and calculates unadjusted prevalence for each group  ttprev["prev_unadj"] <- 0;  ttprev$prev_unadj <- (ttprev$tt / ttprev$RESIDENTS)  *#generate unadjusted EU-level prevalence for comparison purposes*  ttprev_unadjusted_cluster <- aggregate(cbind(RESIDENTS, tt) ~ EU+CLUSTER, data=ttprev, sum)  ttprev_unadjusted_cluster$cluster_prev_unadj <- (ttprev_unadjusted_cluster$tt/ttprev_unadjusted_cluster$RESIDENTS)  ttprev_unadjusted <- aggregate(cluster_prev_unadj ~ EU, data=ttprev_unadjusted_cluster, mean)  colnames(ttprev_unadjusted)[colnames(ttprev_unadjusted)=="cluster_prev_unadj"] <- "ttprev_unadj"  #This creates the new column named "adj_tt" filled with zeros and Iights the data  ttprev["adj_tt"] <- 0;  ttprev$adj_tt <- (ttprev$prev_unadj * ttprev$age_Iight)  *#collapse on cluster level and get sum of Iighted prevalence*  ttprev_cluster <- aggregate(adj_tt ~ EU + CLUSTER, data = ttprev, sum)  *#calculate mean of adjusted cluster prevalences*  ttprev_eu <- aggregate(adj_tt ~ EU, data = ttprev_cluster, mean)  a <- merge(ttprev_unadjusted, ttprev_eu, by="EU", all=TRUE)  *##bootstrap to generate confidence intervals*  dataset <- ttprev_cluster  str(dataset)  dataset$EU <- as.factor(dataset$EU)  dataset$cluster <- as.factor(dataset$CLUSTER)  dataset$cluster_prev <- dataset$adj_tt  *#boot statistic function*  clustermean <- function(df, i) {  num_clusters <- nrow(df)  r <- round(runif(num_clusters, 1, nrow(df))) #nrow(df) allows the analysis to divide by the correct #clusters  df2 <- numeric()  for (i in 1:num_clusters) {  df2[i] <- df[r[i],]$cluster_prev  }  return(mean(df2))  }  *#create empty data frame for results*  bootResult_tt <- data.frame(EU=character(), bootmean=numeric(), se=numeric(), ci95_low=numeric(), ci95_high=numeric(), stringsAsFactors=FALSE)  *#bootstrap function, looped over each EU*  library(boot)  num_reps <- 10000 #Should be at least 1000 but preferably 10000, higher reps the more precise  for (i in 1:nlevels(dataset$EU)) {  data2 <- subset(dataset, EU==levels(EU)[i])  b <- boot(data2, clustermean, num_reps)  m <- mean(b$t)  se <- sd(b$t)    *#calculate 2.5/97.5 percentiles as Confidence Interval*  q <- quantile(b$t, c(0.025, 0.975))  ci_loIr <- q[1]  ci_upper <- q[2]    *#write result to data frame*  eu_temp <- as.character(data2$EU[1])  bootResult_tt[i,] <- c(eu_temp, m, se, ci_loIr, ci_upper)    *#histogram of mean bootstrap results with CI*  hist(b$t, breaks=50, main=paste("EU",eu_temp, "- Histogram of mean bootstrap results, n=", num_reps))  abline(v=ci_loIr, lty="dashed", col="black" )  abline(v=ci_upper, lty="dashed", col="black" )  #abline(v=ci_loIrSE, lty="dashed", col="blue" )  #abline(v=ci_upperSE, lty="dashed", col="blue" )  }  *#merge prevalence and confidence intervals into one table*  b <- merge(ttprev_unadjusted, ttprev_eu, by="EU", all=TRUE)  b <- merge(b,bootResult_tt, by="EU", all=TRUE)  results_bootstrap <- merge(b,bootResult_tt, by="EU", all=TRUE)  *#write combined results to csv file*  write_filename <- "Results.csv"  write.csv(results_bootstrap, write_filename) |
| --- |
