# Supplementary material for: The global burden of trichiasis in 2016
Source: PLoS Negl Trop Dis. 2019 Nov 25;13(11):e0007835. doi: 10.1371/journal.pntd.0007835 (PMC6901231; doi:10.1371/journal.pntd.0007835)
Supplement: S2 Appendix — (DOCX) [file pntd.0007835.s002.docx]

##### TT backlog sensitivity analysis

| Country | Location | Hygiene | | | Sanitation | | | | Population | TT backlog estimate |
| --- | --- | --- | --- | --- | --- | --- | --- | --- | --- | --- |
|  |  | Basic service | Limited service | No handwashing facility | At least basic | Limited service | Open defecation | Unimproved |  |  |
| Angola | rural | 15% | 12% | 73% | 21% | 5% | 56% | 17% |  |  |
|  | urban | 37% | 13% | 50% | 62% | 27% | 3% | 7% |  |  |
|  | total | 25% | 12% | 63% | 39% | 15% | 33% | 13% | 25,789,024 | 3,138 |
| Zambia | rural | 5% | 24% | 71% | 19% | 7% | 25% | 50% |  |  |
|  | urban | 26% | 33% | 41% | 49% | 20% | 1% | 30% |  |  |
|  | total | 14% | 28% | 59% | 31% | 12% | 15% | 41% | 12,526,314 | 1,524 |
|  |  |  |  |  |  |  |  |  |  |  |
| Country | Location | Hygiene | | | Sanitation | | | | Population | TT backlog estimate |
|  |  | Basic service | Limited service | No handwashing facility | At least basic | Limited service | Open defecation | Unimproved |  |  |
| Botswana | rural |  |  |  | 39% | 10% | 36% | 14% |  |  |
|  | urban |  |  |  | 75% | 6% | 2% | 16% |  |  |
|  | total |  |  |  | 60% | 8% | 17% | 15% | 2,024,904 | 1,049 |
| Zimbabwe | rural | 24% | 52% | 25% | 31% | 15% | 39% | 15% |  |  |
|  | urban | 46% | 38% | 16% | 54% | 42% | 0% | 4% |  |  |
|  | total | 31% | 47% | 22% | 39% | 24% | 26% | 11% | 13,061,239 | 6,765 |
|  |  |  |  |  |  |  |  |  |  |  |
| Country | Location | Hygiene | | | Sanitation | | | | Population | TT backlog estimate |
|  |  | Basic service | Limited service | No handwashing facility | At least basic | Limited service | Open defecation | Unimproved |  |  |
| Burundi | rural |  |  |  | 51% | 6% | 3% | 39% |  |  |
|  | urban |  |  |  | 46% | 40% | 1% | 13% |  |  |
|  | total |  |  |  | 50% | 11% | 3% | 36% | 7,877,728 | 155 |
| Uganda | rural | 6% | 22% | 72% | 17% | 9% | 7% | 67% |  |  |
|  | urban | 15% | 21% | 64% | 28% | 43% | 2% | 27% |  |  |
|  | total | 8% | 22% | 71% | 19% | 14% | 6% | 60% | 34,634,650 | 680 |

|  |  |  |  |  |  |  |  |  |  |  |
| --- | --- | --- | --- | --- | --- | --- | --- | --- | --- | --- |
| Country | Location | Hygiene | | | Sanitation | | | | Population | TT backlog estimate |
|  |  | Basic service | Limited service | No handwashing facility | At least basic | Limited service | Open defecation | Unimproved |  |  |
| Namibia | rural | 27% | 58% | 15% | 15% | 3% | 76% | 5% |  |  |
|  | urban | 62% | 28% | 9% | 55% | 21% | 20% | 4% |  |  |
|  | total | 44% | 44% | 12% | 34% | 11% | 50% | 5% | 2,113,077 | 1,094 |
| Zimbabwe | rural | 24% | 52% | 25% | 31% | 15% | 39% | 15% |  |  |
|  | urban | 46% | 38% | 16% | 54% | 42% | 0% | 4% |  |  |
|  | total | 31% | 47% | 22% | 39% | 24% | 26% | 11% | 13,061,239 | 6,765 |
|  |  |  |  |  |  |  |  |  |  |  |
| Country | Location | Hygiene | | | Sanitation | | | | Population | TT backlog estimate |
|  |  | Basic service | Limited service | No handwashing facility | At least basic | Limited service | Open defecation | Unimproved |  |  |
| Iran  (Islamic Republic of) | rural |  |  |  | 79% | 17% | 2% | 3% |  |  |
|  | total |  |  |  | 88% | 10% | 1% | 1% | 75,149,669 | 1,928 |
|  | urban |  |  |  | 92% | 7% | 0% | 1% |  |  |
| Pakistan | rural | 46% | 43% | 11% | 48% | 9% | 19% | 24% |  |  |
|  | total | 60% | 31% | 8% | 58% | 8% | 12% | 22% | 207,774,520 | 5,330 |
|  | urban | 83% | 12% | 5% | 74% | 8% | 0% | 18% |  |  |
|  |  |  |  |  |  |  |  |  |  |  |
| Country | Location | Hygiene | | | Sanitation | | | | Population | TT backlog estimate |
|  |  | Basic service | Limited service | No handwashing facility | At least basic | Limited service | Open defecation | Unimproved |  |  |
| Iraq | rural | 81% | 7% | 12% | 60% | 9% | 0% | 5% |  |  |
|  | total | 91% | 4% | 5% | 54% | 10% | 0% | 4% | 19,184,543 | 492 |
|  | urban | 95% | 2% | 2% | 51% | 11% | 0% | 3% |  |  |
| Pakistan | rural | 46% | 43% | 11% | 48% | 9% | 19% | 24% |  |  |
|  | total | 60% | 31% | 8% | 58% | 8% | 12% | 22% | 207,774,520 | 5,330 |
|  | urban | 83% | 12% | 5% | 74% | 8% | 0% | 18% |  |  |

|  |  |  |  |  |  |  |  |  |  |  |
| --- | --- | --- | --- | --- | --- | --- | --- | --- | --- | --- |
| Country | Location | Hygiene | | | Sanitation | | | | Population | TT backlog estimate |
|  |  | Basic service | Limited service | No handwashing facility | At least basic | Limited service | Open defecation | Unimproved |  |  |
| Mexico | rural | 80% | 15% | 5% | 81% | 8% | 6% | 6% |  |  |
|  | total | 88% | 9% | 3% | 44% | 7% | 2% | 2% | 112,336,538 | 5,428 |
|  | urban | 90% | 8% | 2% | 45% | 7% | 1% | 1% |  |  |
| Guatemala | rural | 70% | 27% | 3% | 53% | 7% | 10% | 30% |  |  |
|  | total | 77% | 21% | 3% | 67% | 9% | 6% | 18% | 11,237,196 | 543 |
|  | urban | 83% | 14% | 2% | 81% | 10% | 1% | 8% |  |  |
|  |  |  |  |  |  |  |  |  |  |  |
| Country | Location | Hygiene | | | Sanitation | | | | Population | TT backlog estimate |
|  |  | Basic service | Limited service | No handwashing facility | At least basic | Limited service | Open defecation | Unimproved |  |  |
| Nauru | total |  |  |  | 66% | 31% | 3% | 1% | 10,084 | 1 |
|  | urban |  |  |  | 66% | 31% | 3% | 1% |  |  |
| Vanuatu | rural |  |  |  | 51% | 13% | 2% | 34% |  |  |
|  | total |  |  |  | 53% | 18% | 2% | 27% | 515,870 | 48 |
|  | urban |  |  |  | 61% | 32% | 1% | 6% |  |  |
