# Supplementary material for: The global burden of trichiasis in 2016
Source: PLoS Negl Trop Dis. 2019 Nov 25;13(11):e0007835. doi: 10.1371/journal.pntd.0007835 (PMC6901231; doi:10.1371/journal.pntd.0007835)
Supplement: S1 Checklist — (DOCX) [file pntd.0007835.s003.docx]

| **STROBE Statement – Checklist of items that should be included in reports of observational studies** | | | |
| --- | --- | --- | --- |
|  | **Item No** | **Recommendation** | **Manuscript Section** |
| Title and abstract | 1 | (*a*) Indicate the study’s design with a commonly used term in the title or the abstract | Abstract  (paragraph 2) |
|  |  | (*b*) Provide in the abstract an informative and balanced summary of what was done and what was found | Abstract  (paragraph 2) |
| **Introduction** | | | |
| Background/rationale | 2 | Explain the scientific background and rationale for the investigation being reported | Background  (paragraph 5-8) |
| Objectives | 3 | State specific objectives, including any prespecified hypotheses | Background  (paragraph 9) |
| **Methods** | | | |
| Study design | 4 | Present key elements of study design early in the paper | Methodology  (paragraph 1-4) |
| Setting | 5 | Describe the setting, locations, and relevant dates, including periods of recruitment,  exposure, follow-up, and data collection | Methodology  (paragraph 8) |
| Participants | 6 |  | NA |
| Variables | 7 | Clearly define all outcomes, exposures, predictors, potential confounders, and effect  modifiers. Give diagnostic criteria, if applicable | Methodology  (paragraph 6) |
| Data sources/measurement | 8* | For each variable of interest, give sources of data and details of methods of assessment (measurement). Describe comparability of assessment methods if there is more than one group | Methodology  (paragraph 2-5) |
| Bias | 9 | Describe any efforts to address potential sources of bias | Methodology  (paragraph 7) |
| Study size | 10 | Explain how the study size was arrived at | NA |
| Quantitative variables | 11 | Explain how quantitative variables were handled in the analyses. If applicable,  describe which groupings were chosen and why | Methodology  (paragraph 7-9) |
| Statistical methods | 12 | (*a*) Describe all statistical methods, including those used to control for confounding | Methodology  (paragraph 7-9) |
|  |  | (*b*) Describe any methods used to examine subgroups and interactions | NA |
|  |  | (*c*) Explain how missing data were addressed | Methodology  (paragraph 9) |
|  |  | (*d*) Describe any sensitivity analyses | Methodology  (paragraph 5-10) |
| **Results** | | | |
| Participants | 13* | (a) Report numbers of individuals at each stage of study—eg numbers potentially eligible,  examined for eligibility, confirmed eligible, included in the study, completing follow-up, and analysed | Results  (paragraph 1) |
| Descriptive data | 14* | (a) Give characteristics of study participants (eg demographic, clinical, social) and information on exposures and potential confounders | Results  (paragraph 1) |
|  |  | (b) Indicate number of participants with missing data for each variable of interest | Results  (paragraph 1) |
| Outcome data | 15* |  | Results  (paragraph 1) |
| Main results | 16 | (*a*) Give unadjusted estimates and, if applicable, confounder-adjusted estimates and their precision (eg, 95% confidence interval). Make clear which confounders were adjusted for and why they were included | Results  (table 1-2) |
| Other analyses | 17 | Report other analyses done—eg analyses of subgroups and interactions, and sensitivity  analyses | Results  (table 2) |
| **Discussion** | | | |
| Key results | 18 | Summarise key results with reference to study objectives | Discussion  (paragraph 1) |
| Limitations | 19 | Discuss limitations of the study, taking into account sources of potential bias or imprecision. Discuss both direction and magnitude of any potential bias | Discussion  (paragraph 3) |
| Interpretation | 20 | Give a cautious overall interpretation of results considering objectives, limitations, multiplicity  of analyses, results from similar studies, and other relevant evidence | Discussion  (paragraph 8) |
| Generalisability | 21 | Discuss the generalisability (external validity) of the study results | Discussion  (paragraph 8) |
